# A Cellular Mechanism of Learning-Induced Enhancement of Synaptic Inhibition: PKC-Dependent Upregulation of KCC2 Activation

**DOI:** 10.1038/s41598-020-57626-2

**Published:** 2020-01-22

**Authors:** Adi Kfir, Richa Awasthi, Sourav Ghosh, Sankhanava Kundu, Blesson Paul, Raphael Lamprecht, Edi Barkai

**Affiliations:** Sagol Department of Neurobiology, Faculty of Natural Sciences, University of Haifa, Haifa, Israel

**Keywords:** Learning and memory, Neurophysiology

## Abstract

Long-term memory of complex olfactory learning is expressed by wide spread enhancement in excitatory and inhibitory synaptic transmission onto piriform cortex pyramidal neurons. A particularly interesting modification in synaptic inhibition is the hyperpolarization of the reversal potential of the fast post synaptic inhibitory potential (fIPSP). Here we study the mechanism underlying the maintenance of such a shift in the fIPSP. Blocking of the neuronal specific K^+^-Cl^−^ co-transporter (KCC2) in neurons of trained rats significantly depolarized the averaged fIPSP reversal potential of the spontaneous miniature inhibitory post synaptic currents (mIPSCs), to the averaged pre-training level. A similar effect was obtained by blocking PKC, which was previously shown to upregulate KCC2. Accordingly, the level of PKC-dependent phosphorylation of KCC2, at the serine 940 site, was significantly increased after learning. In contrast, blocking two other key second messenger systems CaMKII and PKA, which have no phosphorylation sites on KCC2, had no effect on the fIPSP reversal potential. Importantly, the PKC inhibitor also reduced the averaged amplitude of the spontaneous miniature excitatory synaptic currents (mEPSCs) in neurons of trained rats only, to the pre-training level. We conclude that learning-induced hyper-polarization of the fIPSP reversal potential is mediated by PKC-dependent increase of KCC2 phosphorylation.

## Introduction

The notion that synaptic inhibition has a central role in memory formation and maintenance has been gaining strong support by recent studies^1–6^. Learning-induced modulation of inhibitory synaptic transmission was shown in several brain areas, following different training paradigms^2,7–11^. It has been suggested that long-lasting enhancement in synaptic inhibition is required to allow long-term memory maintenance while preventing uncontrolled hyper-excitability^5,12–14^.

The mechanisms underlying learning-induced and activity-induced plasticity of synaptic inhibition have been the subject of an increasing number of studies^9,15–17^. We previously showed that complex olfactory-discrimination (OD) learning results in a long-lasting enhancement of GABA_A_-mediated inhibitory synaptic transmission in piriform cortex pyramidal neurons, which is widely spread throughout the piriform cortex pyramidal cell population^9,11,13,14,16,18^.

Such learning-induced enhanced inhibition is mediated by two mechanisms; increased single-channel conductance of the GABA_A_-channel receptor^11,13^ and a hyperpolarizing shift in the chloride current mediated fIPSP reversal potential,^9,18^. Learning-induced enhancement of the GABA_A_ channel conductance is maintained for days by persistent CaMKII activation^11^. The molecular mechanism that enables long-lasting hyperpolarization of the fIPSP reversal potential is yet to be explored.

The chloride reversal potential is determined to a great extent by the combined action of two co-transporters; the Na^+^-K^+^, 2Cl^−^ co-transporter (NKCC1) increases intracellular Cl^−^ concentration, thus depolarizing the reversal potential of the GABA_A_-mediated current, while the neuron specific K^+^-Cl^−^ co-transporter (KCC2) extrudes Cl^−^, thereby hyperpolarizing the reversal potential of the same current (for review see^19^). The cation-chloride co-transporters, particularly KCC2, have been implicated in neuronal plasticity^20^. Activity-induced modulation of KCC2 which result in change of the GABA_A_-current reversal potential was shown in hippocampal neurons^21,22^. Thus, learning-induced modulation of the KCC2 activity is a plausible explanation for long-term hyperpolarization of the Cl^−^ reversal potential.

Notably, in the above mentioned studies, neuronal activity resulted in reduced KCC2 activity and thus in decrease of the GABA_A_-mediated fast synaptic inhibition. Enhanced KCC2 activity, which should result in increase of this synaptic current, can be induced by phosphorylation of the serine 940 residue on the cytoplasmic C-terminal domain^23–25^.

Here we show that learning-induced hyperpolarization of the reversal potential of the fast GABA_A_-mediated fast synaptic inhibition is maintained by enhanced KCC2 activation and requires persistent activation of PKC for its long-term maintenance.

## Results

**Figure 1:**
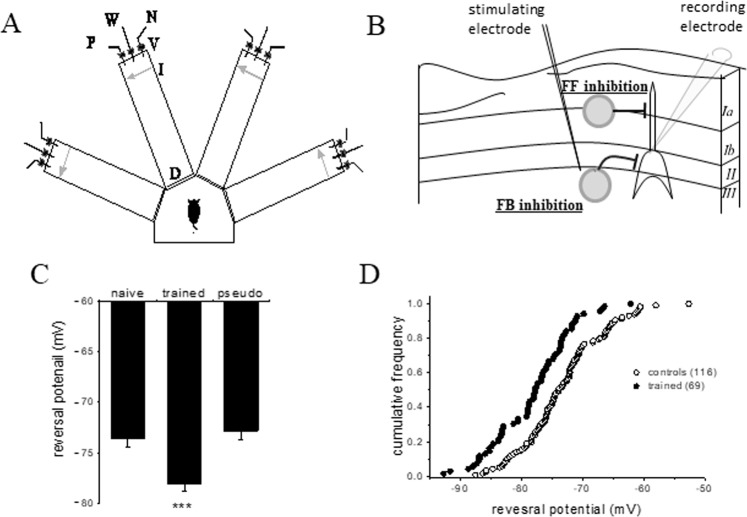
Complex olfactory learning-induced hyperpolarization of the fast IPSP. (**A**) Schematic description of the olfactory maze. An electronic ‘start’ command opens randomly two out of eight valves (V), releasing a positive-cue odor (P) into one of the arms and a negative-cue odor (N) into another. Eight seconds later, the two corresponding guillotine doors (D) are lifted to allow the rat to enter the selected arms. Upon choosing the arm containing the positive-cue odor, reaching the far end of an arm (90 cm long), the rat body interrupts an infrared beam (I, arrow) and a drop of drinking water is released from a water hose (W) into a small drinking well. A trial ends when the rat interrupts a beam, or in 10 seconds, if no beam is interrupted. A fan is operated for 15 seconds between trials, to remove odors. (**B**) Schematic illustration of the piriform cortex in a coronal brain slice and the experimental procedure. Intracellular recordings were performed from cell bodies in layer II. Feed forward inhibition (**FF**) inputs are terminated on the distal apical dendrites, while feedback inhibition (**FB**) is terminated on cell bodies and proximal dendrites of the cell^41,42^. For activation of inhibitory synaptic inputs, electrical stimuli were applied at the border between layers II and III, in the presence of the glutamatergic AMPA and NMDA receptors blockers. (**C**) Learning-induced hyperpolarization of the fast IPSP’s reversal potential. Averaged values of the IPSP’s reversal potential in the three experimental groups. This value is significantly hyperpolarized for the neurons taken from trained rats (***p < 0.001). (**D**) A cumulative frequency graph comparing the reversal potentials of the fast IPSP in neurons from controls versus trained rats. Each point represents *V*_Cl,_ the reversal potential of the fast IPSP for a neuron. Notably, the curve for the trained group is shifted smoothly leftwards, indicating that learning-induced reduction in the fIPSP reversal potential is apparent throughout the recorded pyramidal neurons population. Data was taken from 50 naive rats, 54 trained rats, and 45 pseudo-trained rats. Values represent mean ± SE.

As previously shown (9 19), the averaged reversal potential of fIPSP was significantly hyperpolarized in neurons from trained rats (−78.1 ± 6.9 mV (n = 69)), compared to naive (−73.6 ± 6.9 mV, n = 65) and pseudo-trained (−72.8 ± 6.4 mV, n = 51) (F = _2,159_, p < 0.001) (Fig. 1C). Neurons from pseudo trained and naïve rats had an identical averaged reversal potential for the early IPSP, and thus values obtained in neurons from these two groups were combined to a single control group (averaged value of 73.3 ± 6.7, n = 116). Such hyperpolarization of the early IPSP reversal potential in neurons from trained rats is apparent throughout the recorded neuronal population, as shown in the cumulative frequency graph (Fig. 1D).

### Learning-induced shift of the fast IPSP reversal potential is abolished by KCC2 inhibition

To examine whether the long-lasting reduction in the fIPSP reversal potential is dependent on enhanced activation of the KCC2 co-transporter, we applied the specific KCC2 inhibitor VU0463271 (10 µM) and tested its effect on the reversal potential of inhibitory synaptic inhibitory potentials (IPSPs) with intracellular sharp electrodes recordings, and the reversal potential of inhibitory synaptic currents (IPSCs) with whole cell patch clamp electrodes recordings.

**Figure 2:**
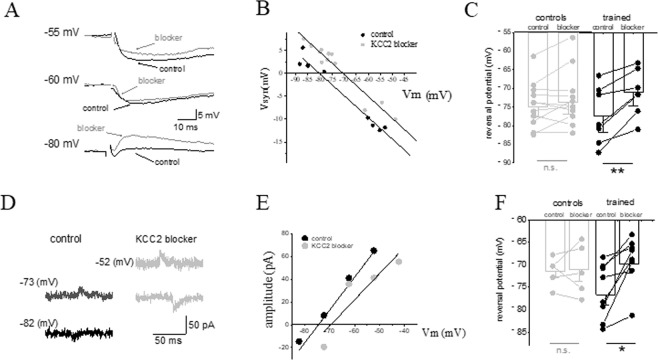
Learning-induced hyperpolarization of the fIPSP and IPSC is mediated by KCC2 upregulation. (**A**) Example of the effect of the KCC2 blocker, VU0463271 on the fIPSP in a neuron of a trained rat. Amplitude of the fIPSP was recorded at several holding potentials. IPSPs were measured at the first peak, as appeared in the most depolarized recording potential. Numbers on the left of the traces note the holding membrane potential. These measurements were then used to calculate the reversal potential of the responses.(**B**) The reversal potentials of the synaptic responses shown in A were determined by the linear regression line, describing the amplitude of the synaptic potential as a function of the membrane holding potential. Application of VU0463271 resulted in a 10 mV depolarization of the fIPSP reversal potential. Note that the slope of the curve is not modified by the KCC2 blocker application, indicating that the conductance of the chloride current via the GABA_A_-channel is not affected by the blocker. (**C**) The averaged reversal potential of the fast IPSP is reduced in neurons from trained rats only. (*P < 0.05 for trained neurons before and after VU0463271 application). Data was taken from 7 trained rats and 10 control rats. Values represent mean ± SE. (**D**) Example of the effect of the KCC2 blocker, VU0463271 on the spontaneous IPCSs in a neuron taken from a trained rat. Amplitude of the IPSCs was recorded at several holding potentials. Averaged amplitude of the IPSC at each membrane potential was calculated by averaging the amplitude of all recorded IPSCs, as previously described^11^. Numbers on the left of the traces note the holding membrane potential. These measurements were then used to calculate the reversal potential of the responses. (**E**) The reversal potentials of the synaptic responses shown in D were determined by the linear regression line, describing the amplitude of the synaptic current as a function of the membrane holding potential. Application of VU0463271 resulted in a 5 mV depolarization of the IPSC reversal potential. (**F**) The averaged reversal potential of the IPSC is reduced in neurons from trained rats only. (**P < 0.01 for trained neurons before and after VU0463271 application). Data was taken from 7 trained rats and 5 control rats. Values represent mean ± SE.

With intracellular sharp electrodes recordings, VU0463271 significantly depolarized the early IPSP reversal potential in neurons from trained rats only. A typical example of the effect of the inhibitor on a neuron from a trained rat is shown in Fig. 2A,B. In neurons from trained rats the averaged reversal potential value was significantly (t = 4.77, P < 0.01, paired t-test) depolarized in VU0463271 (from to 77.3 mV ± 7.7 in control conditions, to 71.1 mV ± 6.2 (n = 7) in VU0463271). For controls the averaged value was similar (t = 1.52, P = 0.16, paired t-test) to that measured without the blocker (from to 74.3 mV ± 6.1 in control conditions, to 72.8 mV ± 7.4 (n = 11) in VU0463271) (Fig. 2C).

With whole cell patch clamp recordings, VU0463271 significantly depolarized the early IPSC reversal potential in neurons from trained rats only. A typical example of the effect of the inhibitor on a neuron from a trained rat is shown in Fig. 2D,E. In neurons from trained rats the averaged reversal potential value was significantly (t = 4.05, P < 0.01, paired t-test) depolarized in VU0463271 (from to 76.7 mV ± 6.1 in control conditions, to 70.6 mV ± 6.0 (n = 8) in VU0463271). For controls, the averaged value was similar (t = 0.22, P = 0.84, paired t-test) to that measured without the blocker (from to 71.5 mV ± 3.2 in control conditions, to 71.1 mV ± 5.8 (n = 5) in VU0463271) (Fig. 2F). Thus, here too in the presence of the KCC2 blocker, the averaged value of the early IPSP reversal potential was similar in neurons from trained and control rats (Fig. 2F).

### Learning-induced shift of the early IPSP reversal potential is abolished by PKC inhibition

**Figure 3:**
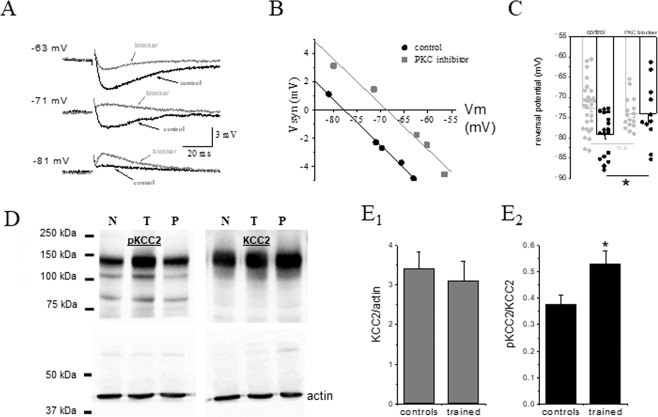
Learning-induced hyperpolarization of the fIPSP is mediated by persistent PKC activation. (**A**) Example of the effect of the PKC blocker, GF-109203×, on the fIPSP in a neuron taken from a trained rat. Amplitude of the fIPSP was recorded at several holding potentials. IPSPs were measured at the first peak, as appeared in the most depolarized recording potential. Numbers on the left of the traces note the holding membrane potential. These measurements were then used to calculate the reversal potential of the responses. (**B**) The reversal potentials of the synaptic responses shown in A were determined by the linear regression line, describing the amplitude of the synaptic potential as a function of the membrane holding potential. Application of GF-109203X resulted in a 9 mV depolarization of the fIPSP reversal potential. Here too, the slope of the curve is not modified by the PKC blocker application. (**C**) The averaged reversal potential of the fIPSP is reduced in neurons from trained rats only (*P < 0.05 for trained neurons before and after GF-109203X application). Data was taken from 15 trained (dark bars) rats and 20 control rats (light bars). Values represent mean ± SE. (**D**) Representative blots of actin, KCC2 and phosphorylated KCC2 (from single blot), in piriform cortex homogenates. Bands represent KCC2 monomer^43^. Blot were cut into two from 70kDa-250kDa and 70 kDa −37kDa. The upper blots were subjected to pKCC2 and KCC2 antibodies, while the lower blots were subjected to actin antibody. (N = naïve, T = trained, P = pseudo trained). (**E**) The expression level of KCC2 compared with actin is not modified after learning (E_1_), while phosphorylation of KCC2 is significantly increased (E_2_). Values represent mean ± SE. (*p < 0.05).

To examine whether KCC2-dependent long-lasting reduction in the fIPSP reversal potential is also dependent on persistent activation of PKC, we applied the specific PKC inhibitor GF-109203X (10 µM). GF-109203X significantly depolarized the fIPSP reversal potential in neurons from trained rats only. A typical example of the effect of the inhibitor on a neuron from a trained rat is shown in Fig. 3A,B. In neurons from trained rats the averaged reversal potential value was significantly (t = 2.10, P < 0.05) depolarized in GF-109203X (from to 79.1 mV ± 5.1 (n = 18) in control conditions, to 73.9 mV ± 7.1 (n = 11) in GF-109203X) (Fig. 3C). For controls the averaged value was similar to that measured without the blocker (from to 72.5 mV ± 5.8 (n = 31) in control conditions, to 73.9 mV ± 4.0 (n = 16) in GF-109203X) (Fig. 2C). Consequently, in the presence of the PKC blocker, the averaged value of the early IPSP reversal potential was similar in neurons from trained and control rats (Fig. 3C).

### PKC dependent phosphorylation of KCC2 is enhanced after learning

Since learning-induced hyperpolarization of the GABA_A_ reversal potential is dependent on both KCC2 activation and persistent PKC activation, we hypothesized that learning should be accompanied by an increased PKC-dependent phosphorylation of the KCC2 cotransporter. Serine 940 is the only known residue of KCC2 whose phosphorylation enhances the co-transporter’s activity^24^. This residue is phosphorylated by PKC^23^.

Using Western blot analysis, we measured what is the percentage of phosphorylated KCC2 in piriform cortex of control and trained animals at the serine 940 site. Measurements were made at two time points after learning. One at the fifth day after rule learning, when learning-induced enhancement of synaptic inhibition is apparent^11,16^ and the second on the fourth day after learning.

As shown in Fig. 3D–E, on the fifth day after learning, the level of KCC2, normalized to the actin levels, was not modified after learning; Averaged values of KCC2/actin were 3.408 ± 2.56 (n = 14) for controls and 3.097 ± 1.68 (n = 7) for trained (t = 0.662, p = 0.44). In contrast, the level of phosphorylated KCC2 was significantly increased (t = 2.589, p = 0.018) after learning compared with controls. Averaged values of pKCC2/KCC2 were 0.378 ± 0.122 for controls (n = 14) and 0.530 + 0.129 for trained (n = 7). Thus, OD learning results in an increase of 40% in the fraction of PKC- dependent phosphorylation of KCC2. On the fourth day after learning averaged values of KCC2/actin were 2.685 ± 0.98 (n = 12) for controls and 2.482 ± 0.809 (n = 6) for trained (t = 0.438, p = 0.67). At this time point the level of phosphorylated KCC2 was not increased (t = 1.529, p = 0.14) after learning compared with controls. Averaged values of pKCC2/KCC2 were 0.317 ± 0.131 for controls (n = 12) and 0.403 + 0.122 for trained (n = 6). Thus, a significant increase in the phosphorylation level of KCC2 is apparent only on the fifth days after learning.

### Learning-induced modulation of the early IPSP reversal potential is not dependent on PKA or CaMKII

Since PKA and CaMKII have been shown to affect the amplitude of GABA_A_-mediated currents^26–29^, and CaMKII is necessary for the maintenance of learning-induced enhancement of GABAergic transmission^11^, we tested if these two second messengers also participate in the learning-induced modification in the fast IPSP reversal potential.

**Figure 4:**
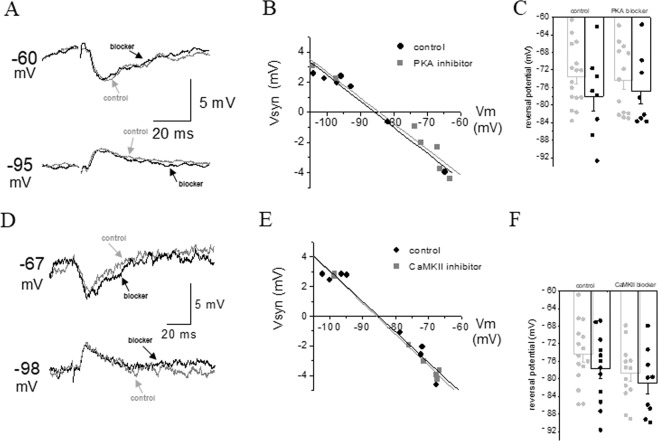
Learning-induced hyperpolarization of the fIPSP is not dependent on PKA or CaMKKI activation. (**A**) Example of the effect of the PKA blocker, H89, on the fIPSP in a neuron taken from a trained rat. Amplitude of the fIPSP was recorded at several holding potentials. IPSPs were measured at the first peak, as appeared in the most depolarized recording potential. Numbers on the left of the traces note the holding membrane potential. These measurements were then used to calculate the reversal potential of the responses. (**B**) The reversal potentials of the synaptic responses shown in A were determined by the linear regression line, describing the amplitude of the synaptic potential as a function of the membrane holding potential. Application of H89 had no effect on the fIPSP reversal potential. (**C**) The averaged reversal potential of the fIPSP in neurons from trained and control rats is not modified by H89 application. Data were taken from 7 trained rats (dark bars) and 11 control rats (light bars). Values represent mean ± SE. (**D**) Example of the effect of the cell-penetrating peptide inhibitor, *tat*CN21, on the fIPSP in a neuron taken from a trained rat. Amplitude of the fIPSP was recorded at several holding potentials. IPSPs were measured at the first peak, as appeared in the most depolarized recording potential. Numbers on the left of the traces note the holding membrane potential. These measurements were then used to calculate the reversal potential of the responses. (**E**) The reversal potentials of the synaptic responses shown in D were determined by the linear regression line, describing the amplitude of the synaptic potential as a function of the membrane holding potential. Application of *tat*CN2 had no effect on the fIPSP reversal potential. (**F**) The averaged reversal potential of the fIPSP is in neurons from trained and control rats is not modified by *tat*CN2 or KN93 application. Data was taken from 9 trained rats (dark bars) and 14 control rats (light bars). Values represent mean ± SE.

As shown in Fig. 4(A–C), application of the specific PKA blocker H89 dihydrochloride (50 µM), did not affect the averaged amplitude of the fast IPSP in neurons from trained rats (78.1 mV ± 9.5 (n = 8) in control conditions, 76.9 ± 8.1 (n = 8) in H89 (t = 0.27, p = 0.79) and in neurons from controls (73.5 mV ± 6.7 (n = 17) in control conditions, 74.3 ± 7.8 (n = 13) in H89 (t = 0.31, p = 0.76)).

To block CaMKII activity, we applied of the cell-penetrating peptide inhibitor, *tat*CN21 (5 µM) or the specific blocker KN93 (10 µM). Application of either one of the inhibitors did not affect the averaged amplitude of the fast IPSP in neurons from trained rats (77.7 mV ± 7.9 (n = 12) in control conditions, 81.0 ± 8.1 (n = 9) with blocker (results of using these two blockers are combined), (t = 0.97, p = 0.35), and in neurons from controls (74.4 mV ± 7.2 (n = 15) in control conditions, 78.7 ± 6.3 (n = 13) with blocker (t = 1.65, p = 0.11)) (Fig. 4D–F).

### Persistent PKC activity also maintains long-lasting enhancement of AMPAR-mediated synaptic excitation

In addition to the long-lasting effect of PKC on maintaining learning-induced modulation inhibitory synaptic transmission presented here, we previously showed that such PKC persistent activity is required for maintaining learning-induced enhanced intrinsic neuronal excitability^30^. We thus tested if this second messenger system has also a key role in the third component that controls cellular excitability, synaptic excitation, which is also enhanced after OD learning^16,31^.

**Figure 5:**
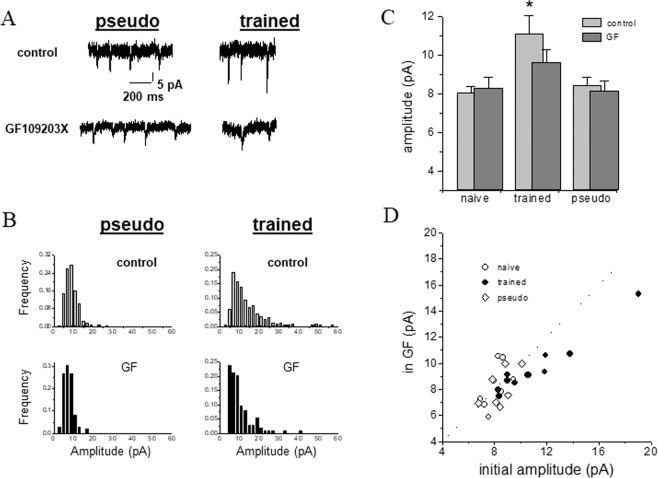
Learning-induced enhancement of spontaneous excitatory events is PKC dependent. (**A**) Spontaneous excitatory events recorded from a pseudo-trained neuron (left) and a trained neuron (right), prior to (top traces) and after (bottom traces) application of the PKC blocker, GF-109203X. While the drug has no apparent effect on the event amplitudes in the pseudo-trained neurons, high amplitude events in the trained neurons were reduced. (**B**) Amplitude histograms of the two neurons recorded in A. Note the large number of high-amplitude events in the trained neuron (top histogram, right) compared with the pseudo-trained neuron (top histogram, left). GF-109203X had little effect on the amplitude distribution in the pseudo-trained neuron (bottom histogram, left), while abolishing most of the high-amplitude events in the trained neuron (bottom histogram, right). (**C**) The averaged amplitude of the excitatory synaptic events is significantly reduced in neurons from trained rats after GF-109203X application (*p < 0.05), as result of which the difference in the average amplitude between the groups disappear. Data taken from 6 naïve, 9 trained and 5 pseudo-trained rats. Values represent mean ± SE. (**D**) GF-109203X reduces mostly the high-amplitude events in the three groups, thus affecting particularly the trained neurons, as seen by the deviation from the dotted line.

Application of GF-109203X (10 µM), reduced significantly (t = 4.02, p < 0.05) the averaged amplitude of the spontaneous excitatory response in neurons from trained rats (Fig. 5A–C). The averaged value of the mEPSCs was reduced from 11.08 ± 3.1 pA to 9.61 ± 2.1 pA (n = 11). The average values of the mEPSCs neurons from naïve rats were 8.07 ± 0.8 pA in control conditions and 8.27 ± 1.7 pA in the GF-109203X (n = 8) and pseudo-trained rats were 8.43 ± 1.07 pA in control conditions and 8.12 ± 1.4 pA in GF-109203X (n = 7). The significant difference in the averaged mEPSC amplitude between the trained and the two control groups before application of the drug (F_2_ = 5.38, p < 0.05) was abolished after GF-109203X treatment (F_2,24_ = 0.14, p = 0.17) (Fig. 5C). GF-109203X abolished mostly the large spontaneous events recorded in trained neurons. The effect of the PKC blocker was most pronounced in neurons from the trained rats (Fig. 5D). The frequency of synaptic events was not affected in by GF- GF-109203X in any of the three groups; the averaged mEPSCs frequency in neurons from naïve neurons was 17.8 ± 9.3 events per minutes before and 19.3 ± 16.8 after blocker application (p = 0.64), in neurons from trained rats the averaged was 35.6 ± 24.4 events per minutes before and 28.9 ± 10.7 (p = 0.32) after blocker application, and in neurons from pseudo-trained rats the averaged was 28.1 ± 21.6 events per minutes before and 26.4 ± 13.5 (p = 0.81) after blocker application.

## Discussion

Training rats in a particularly difficult olfactory-discrimination task results in acquisition of high skill to perform the task superbly^32^. Such high skill acquisition, termed ‘rule learning’ or ‘learning set’, induces of long-term modifications in intrinsic neuronal properties of piriform cortex pyramidal neurons, as well as in their excitatory and inhibitory synaptic inputs^9,16,18,30,32–34^. These changes, while supporting memory maintenance, must be counter balanced by processes that would overcome the tendency to overspreading of activity and uncontrolled strengthening of synaptic connectivity (see for example, 12).

Indeed, such complex olfactory learning results with post synaptic enhancement of synaptic inhibition onto principle excitatory neurons which is mediated by at least two distinct mechanisms; Enhanced single-channel conductance of the post synaptic GABA_A_ receptor^11,14^ and hyperpolarization of the GABA_A_-mediated fast IPSP^9,18^. Enhanced inhibitory synaptic conductance was show to be mediated by persistent CaMKII activation^11,14^. Here we show that the long-lasting modulation of the fIPSP reversal potential is mediated by a different molecular pathway.

### Long-term hyperpolarization of the fast IPSP reversal potential is mediated by enhanced KCC2 activation

The notion that in neurons from mature animals, the intrinsic concentration of chloride ion is controlled mainly by the KCC2 co-transporter gained strong support in the last decade^20,22–25,35,36^ Thus, long-term modulation of KCC2 activation is the primary candidate to mediate learning-induced hyperpolarization of the fast IPSP, which is mediated by chloride current via the GABA_A_-receptor. Our hypothesis was that learning-induced hyperpolarization of the fast IPSP reversal potential results from enhanced KCC2 activity. Indeed, blocking the co-transporter activity abolished the difference in the IPSP reversal potential between neurons for control and trained rats. Notably, previous studies that explored long-term modifications induced by other methods (pathological or activity-induced modifications) in KCC2 activity, reported reduced co-transporter activation, which resulted in depolarized IPSP reversal potential^21,22,37^. Here we show an opposite effect of enhancing KCC2-mediated hyperpolarizing the IPSP reversal potential. Although KCC2 is also present in the pirifom cortex of naïve and pseudo-trained rats, we did not find a significant effect of blocking KCC2 on the reversal potential of the fast IPSP in neurons from control animals. Notably, our data show a small, insignificant hyper-polarizing effect of KCC2 block, which amounts to an average of 1.5 mV in sharp electrodes recordings (p = 0.15). We propose that in control neurons the IPSP reversal potential is close to the resting membrane potential, and thus blocking the KCC2 co-transporter does not affect the IPSP reversal potential for the time of the intracellular recording (up to 45 minutes after blocker application).

### Enhanced KCC2 activity is maintained by persistent PKC phosphorylation

Modulation of KCC2 activity, and subsequently of the reversal potential of the chloride current, by phosphorylation of the co-transporter has been shown in ample studies (reviewed in^19,20,24,25^). In particular, serine 940 is the only known residue of KCC2 whose phosphorylation enhances the co-transporter’s activity^24^. This residue is phosphorylated by PKC^23^. Our data show that blocking PKC activity *in vitro* abolishes the difference in the fast IPST reversal potential between neurons from trained rats and controls. Moreover, the phosphorylation levels of KCC2, at the Serine 940 site, the only site known to be phosphorylated by PKC is increased considerably after learning. Thus we suggest that learning-induced enhanced activity of KCC2 requires persistent PKC activation for its maintenance. Notably, elevated glutamate levels result in dephosphorylation of the serine 940 residue, in a process mediated by NMDA receptor activity and the subsequent calcium influx into the neurons^37^. Such dephosphorylation results in depolarizing GABA_A_-mediated currents^37^ and subsequently to neuronal hyper-excitability and to a series of pathological conditions^25^. Thus, PKC activation is essential also for counterbalancing serine 940 dephosphorylation triggered by high glutamate levels secreted by excitatory neurons during the process of learning.

### CaMKII and PKA do not affect the fast IPSP reversal potential

CaMKII and PKA regulate the post synaptic inhibitory conductance by phosphorylation of specific sites on the GABA_A_-receptor^26^. In particular, CaMKII phosphorylation of the GABA_A_ receptor regulates the expression and function of the cell surface receptors^28,29^. PKA can inhibitory synaptic transmission bi-directionally by differential phosphorylation of two sites on GABA_A_ beta3 subunits^27^. Notably, these two second messenger systems are not implicated in affecting KCC2 activity directly^24,25^. The finding that these two central second messengers, which have been shown to have key roles in most key learning-relevant modifications, are not related to learning-induced hyperpolarization of the fast IPSP lends further support to our conclusion that this particular effect is mediated by enhanced KCC2 activation.

### Persistent PKC activation also maintains learning-induced enhanced excitatory synaptic transmission

Our previous studies show that learning results in a long-term enhancement of the three components that control the cells excitability; intrinsic neuronal excitability, expressed in enhanced spike firing^30,34,38^, excitatory synaptic transmission^16,31^ and inhibitory synaptic transmission^9,11,18^. In particular, persistent PKC activity is essential for the maintenance of enhanced intrinsic excitability^30^. Our results here show that PKC is also essential for maintaining the other two components that control neurons excitability; persistent PKC activation enhances inhibitory synaptic transmission by upregulating KCC2 and also enhances excitatory synaptic transmission by increasing amplitude of the excitatory, AMPA-receptor mediated, currents. These three combined effects position PKC as a major factor in controlling and maybe also coordinating the neuron’s homeostasis, which must be maintained to allow long-term memory storage. Notably, PKC also affects the GABA_A_-mediated currents^39,40^, but this effect is not modulated by learning^11^.

To conclude our data show that OD complex learning induces long-term up-regulation of the KCC2 co-transporter activity, as results of which the GABA_A_-mediated current reversal potential is hyperpolarized. Such a hyperpolarization may contribute to enhance synaptic inhibition efficiency in controlling the neuron’s excitability. In addition, PKC emerges as a key factor in maintaining long-term synaptic and intrinsic modifications in a well-coordinated manner.

## Methods

### Animal training

#### Subjects and apparatus

As previously described^32–34^, age-matched young adult Sprague-Dawly male rats (Envigo RMS, Israel) were used. Only male rats were used since our previous studies on rule-learning induced long-term modulation of synaptic transmission were performed on brains taken from male rats. Prior to training they were maintained on a 23.5 hr water-deprivation schedule, with food available *ad libitum*. Olfactory discrimination training protocol was performed daily on each trained and pseudo-trained rat in a 4-arm radial maze (Fig. 1A), with commercial odors that are regularly used in the cosmetics and food industry^32,34^. Animal experiments were done according to NIH guidelines and approved by the University of Haifa animal use committee.

#### Training

Olfactory training consisted of 20 trials per day for each rat as previously described^32^. In short, in each trial the rat had to choose between two odors (positive- and negative-cue) presented simultaneously. Rats designated to the trained group were rewarded upon choosing the positive cue. Rats in the pseudo-trained group were rewarded in a random fashion, upon choosing any odor. The criterion for learning was at least 80% positive-cue choices in the last 10 trials of a training day, as was previously used^32^. Rats in the naive group were water restricted, but not exposed to the maze. Typically, 2–3 trained rats and 2–3 pseudo-trained rats were trained at the same training period, and all the rats in the trained group had to meet the criteria for the first pair of odors before all trained and pseudo trained rats were exposed to a second pair of odors. Training for a new pair began only after training for the second pair was completed for all rats. Rats were trained with two pairs of odors to ensure rule learning^32,34^.

As previously described^32^, rats indeed learned the second pair of odors much faster than the first pair (7–8 days of training for the first pair and 1–2 days for the second pair). These data confirm our precious observation^32–34^, that there are two phases of olfactory-discrimination learning, each with a distinct time course: a first phase during which the animal gradually acquires the appropriate behavioral strategy for completing the task (rule learning) and a second phase when the subject quickly acquires specific odor/reward associations (pair learning). Pseudo trained rats received that same number of runs^32,34^.

The experimenters who performed the electrophysiological recordings and the western blots were not aware to the identity of the rat from which the slices or blots were taken.

### Electrophysiological recordings

The procedure for intracellular recordings with sharp electrodes and whole-cell patch clamp recordings are described in detail in our previous publications (9, 19, 32–34 for sharp electrodes. 11, 17, 31 for whole cell patch clamp recordings).

### Intracellular recordings with sharp electrodes

Rats were sacrificed five days after training completion, when olfactory learning-induced enhance of inhibitory synaptic transmission in the piriform cortex is at its peak^9,11,16^. Experiments were done blind; the group affiliation of the rats (naive, trained, or pseudo-trained) was unknown to the person conducting the experiments and measurements. Rats were anesthetized with Pentobarbital (60 mg/kg), the brain was removed, and coronal brain slices of 400 μm were cut as previously described^9^. Brain slices were kept in oxygenated (95% O_2_ + 5% CO_2_) artificial cerebro-spinal fluid (ACSF) containing NaCl 124 mM, KCl 3 mM, MgSO_4_ 2 mM, NaH_2_PO_4_ 1.25 mM, NaHCO_3_ 26 mM, CaCl_2_ 2 mM and glucose 10 mM.

Layer II pyramidal neurons receive inhibitory synaptic inputs from two sources; feed-forward (FF) inhibition is received from afferent inputs arriving to layer Ia via GABAergic neurons located at layer I, while feed-back (FB) inhibition is evoked by layer II pyramidal neurons via GABAergic neurons located in layer III. Inhibitory synaptic transmission is morphologically segregated; FF inhibition is terminated on the distal portion of the apical dendrite, while FB inhibition is terminated on the proximal dendrites^41,42^. Consequently, DC current passed via the sharp recording electrode would be much more efficient in modifying the neuron’s membrane potential at the locations where FB inhibition is generated^9,18^. Thus, the stimulation electrode was placed just under layer II, at the border of layer III (Fig. 1B), to evoke only FB inhibition. Synaptic stimuli were applied at a frequency of 0.05 Hz.

Recordings were performed in layer II pyramidal neurons using sharp microelectrodes with 4M K-Acetate containing electrodes as previously described^9,18^. Membrane potential was shifted to different values by applying DC current via the recording electrode. Six to ten synaptic responses were evoked at and digitally averaged to produce the measured response for each membrane potential. The reversal potential of the synaptic response was interpolated with a linear fit of the graph describing the synaptic response as the function of the membrane potential. In each cell, IPSPs were recorded at 5–6 different membrane potentials. The fast IPSP (fIPSP) amplitude was measured at the first peak after stimulation, which usually occurred with a delay of 7–15 ms from synaptic stimulation^9,18^.

### Slice preparation for whole cell patch clamp recordings

Three hundred μm coronal brain slices taken from the posterior piriform cortex were cut as previously described^31^ and kept for at least two hours in oxygenated (95% O_2_ + 5% CO_2_) normal saline Ringers’ (N.S.R.) solution, as described above. Then, slices were placed in a recording chamber under infrared DIC microscope, and perfused with Ringer’s solution at 30 °C. Whole cell voltage-clamp recordings were obtained from visually identified pyramidal neurons in layer II of the piriform cortex. All electrophysiological recordings were performed using Axopatch 1D (Molecular Devices), and the data were acquired using pClamp9 (Molecular Devices).

All experiments were done blind. One or two neurons were recorded from each rat.

### Miniature excitatory post synaptic current (mEPSC) recordings

To compare between populations of cells from different rat groups, we aimed to maintain the cells at physiological conditions and avoided the use of any artificial blockers that might bias the data by variation in blocker effect. Therefore, cells were voltage-clamped at a membrane potential (V_m_) of −80 mV, which is the resting potential in these cells, as previously measured with intracellular current-clamp recordings^32^. At this voltage, most voltage-dependent channels are closed and NMDA receptors are rarely activated.

The recording electrode was filled with a solution containing (in mM) 140 K-gluconate, 1 EGTA, 6 KCl, 4 NaCl, 2MgCl_2_, and 10 HEPES, pH 7.25. Miniature excitatory postsynaptic currents (mEPSCs) were recorded in the presence of 1 μM tetrodotoxin (TTX). At the end of each experiment, additional recording was performed with 20 µM 6, 7-dinitroquinoxaline-2, 3-dione (DNQX) in the perfusing solution, for at least 10 min, to ensure that there was no contamination of the data with non-AMPAR-mediated events. Given the low concentration of chloride in the patch pipette solution (10 mM), the reversal potential of GABA-mediated inhibitory postsynaptic currents (IPSCs) would be around −65 mV, and thus miniature IPSCs (mIPSCs) at V_m_ = −80 mV should be very small^17^. Recordings of spontaneous events were performed 10–15 min after membrane rupture and lasted for up to 45–50 min. Each neuron was recorded at least 15 min previous to and 15 min after drug application. Each drug was applied for at least 20 min before recording its effect on the neuron. Drugs were applied using a gravity-based system, and the exchange solution time is <5 min. For data analysis, each event was detected by eye and measured using the Mini “Analysis” software (Synaptosoft).

### Miniature inhibitory post synaptic current (mIPSC) recordings

Layer II pyramidal neurons receive strong synaptic inhibition from several classes of GABAergic interneurons^41,42^. To record GABA_A_-mediated mIPSCs (Fig. 1B), the recording electrode contained (in mM): 140 Cesium chloride, 1 EGTA, 6 KCl, 4 NaCl, 2 MgCl_2_, and 10 HEPES. PH = 7.25, 280 mOsm. In these conditions the reversal potential of chloride is at the value of about 0 mV, and thus strong GABA_A_-mediated currents can be studied at holding potential of −60 mV. The perfusion solution contained TTX (1 μM), and also DNQX (20 μM) and APV (50 μM), to block glutamatergic synaptic transmission via AMPA receptors, thus allowing recording of pure IPSCs. Recordings of spontaneous events were performed 15 minutes after membrane rupture and lasted for up to 1 hour. Each neuron was recorded previously to and after drug application.

During the continuous recording of excitatory and inhibitory synaptic events, the current response to 200 ms voltage step of −5 mV was monitored at 1 Hz. A change in the response caused exclusion of the data.

### Drug application

The KCC2 blocker VU0463271(Tocris) (10 µM), the PKC blocker GF-109203X (Cayman) (10 µM), the PKA blocker H89 dihydrochloride (Tocris) (50 µM), The CaMKII specific blocker KN93 (Cayman) (10 µM), the cell-CaMKII penetrating peptide inhibitor, *tat*CN21 (GL Biochem. Shanghai) (5 µM), the GABA_A_ blocker BMI Tocris) (20 µM), the AMPA receptors blocker DNQX (Cayman) (0.2–20 µM) and NMDA receptors blocker APV (Sigma) (50 µM) were applied via the medium solution. For experiments were the VU0463271 was applied, each neuron was recorded prior to and after the KCC2 blocker application. Each neuron was exposed 30 minutes to the drug, prior to determining its effect on the IPSPs or IPSCs reversal potential.

### Immunoblot analysis

Immunoblot analysis was performed as described in our previous publications^11^.

Rats from the three groups (naive, trained, and pseudo-trained) were sacrificed on day 5 after the learning set. The whole brain was immediately frozen in liquid nitrogen and kept at −80 °C. Posterior piriform cortices punch outs from both the hemispheres were dissected out in a cryostat and kept at −80 °C until further use.

Tissues were homogenized in ice-cold lysis buffer (in mM: 10 HEPES, 2 EDTA, 2 EGTA, 0.5 DTT, and 4% protease inhibitor cocktail; SIGMA, 1% phosphatase inhibitor cocktail; SIGMA) using a glass Teflon homogenizer. The homogenate was centrifuged at 10,000 g for 15 min at 4 °C, and the supernatant was collected. An equal volume of 2x sample buffer (5 mM Tris-HCl, pH 6.8, 10% glycerol, 2.3% SDS, and 5% β-mercaptoethanol) was added to the lysate that was subsequently heated to 80 °C for 5 min, vortexed, and stored at −80 °C. Ten microliters of tissue extracted from each sample were separated with 10% SDS-PAGE at 25 mA followed by blotting on to a nitrocellulose membrane at 350 mA for 1.5 h. Blots were cut into two from 70kDa-250kDa and from 70 kDa −37 kDa as shown in Fig. 3D.

Blots were blocked in 5% BSA prepared in Tris-buffered saline Tween (TBST) for 1 h at room temperature followed by incubation with a primary antibody (1:1000, Anti-Potassium Chloride Cotransporter (KCC2) pS940(Rabbit) Antibody-612–401-E15, Rockland; 1:1000, rabbit Anti- K/Cl Cotransporter (KCC2) 07–432; Millipore, or 1:1000, rabbit Anti-beta Actin Antibody; Millipore) overnight at 4 °C in a shaker.

The next day, blots were washed five times (5 min for each wash) with TBST. For secondary antibodies, horseradish peroxidase-conjugated, (1∶10,000, anti-mouse immunoglobulin; Invitrogen) and (1∶10,000, anti-goat immunoglobulins; Invitrogen) were used, then blots were incubated at room temperature for 1.5 h. After five more washings with TBST, proteins were visualized by enhanced chemiluminescence EZ-ECL (Biological Industries) and quantified using a CCD camera (XRS, Bio-Rad) at 30 seconds exposure time and Quantity One software.

### Statistical analysis

For the treatment in which all neurons were recorded prior to and after drug application a paired t-test was applied. For other treatments One-way ANOVA was used to evaluate significance of difference between three and more cell populations. Student’s t-test was used to compare between two cell populations. Throughout the manuscript, ‘n’ refers to the number of neurons. One neuron was recorded from most animals. Two neurons were recorded from each rat at the most.

Linear regression was performed with OriginLab software, using weighted least-square method to fit a linear model function to data.

## Data Availability

All materials, data and associated protocols are promptly available to readers without undue qualifications in material transfer agreements.
